# Effects of Autologous Platelet-Rich Fibrin in Post-Extraction Alveolar Sockets: A Randomized, Controlled Split-Mouth Trial in Dogs with Spontaneous Periodontal Disease

**DOI:** 10.3390/ani10081343

**Published:** 2020-08-04

**Authors:** Adolfo Maria Tambella, Francesca Bartocetti, Giacomo Rossi, Livio Galosi, Giuseppe Catone, Annastella Falcone, Cecilia Vullo

**Affiliations:** 1 School of Biosciences and Veterinary Medicine, University of Camerino, via Circonvallazione, 93, 62024 Matelica (MC), Italy; francesca.bartocetti@studenti.unicam.it (F.B.); giacomo.rossi@unicam.it (G.R.); livio.galosi@unicam.it (L.G.); 2 Dipartimento di Scienze Veterinarie, Università degli Studi di Messina, 98168 Messina, Italy; gcatone@unime.it (G.C.); afalcone@unime.it (A.F.); 3 Dipartimento di ChiBioFarAm, Università degli Studi di Messina, 98122 Messina, Italy; cvullo@unime.it

**Keywords:** dog, extraction sockets, periodontal disease, platelet-rich fibrin, regenerative medicine, socket healing, tooth extraction

## Abstract

**Simple Summary:**

The effects of autologous platelet-rich fibrin were evaluated in dogs with spontaneous periodontal disease after tooth extraction. Both radiographic and histological findings attributed to the platelet-rich fibrin a potential ability to stimulate the natural process of tissue healing and regeneration of bone and soft tissues. Platelet-rich fibrin could, therefore, be considered as a simple and effective therapeutic aid in the management of post-extraction socket healing in dogs.

**Abstract:**

Periodontal disease (PD) is a common inflammatory condition in dogs; in severe stages, dental extraction is frequently required. Platelet-rich fibrin (PRF) has been used in human oral surgical procedures and has been experimentally tested on post-extraction sockets in healthy dogs. This is the first split-mouth, randomized, controlled trial designed to compare post-extractive alveolar socket healing with and without topical application of PRF in canine spontaneous PD. Clinical evaluation, radiographic density, and histological scores for inflammation and regeneration were assessed at recruitment (T0) and after a three-week follow up (T1) on 12 dogs, for a total of 31 pairs of sockets. No complications or clinically evident differences between the treated sites and the control sites were observed. Comparing the radiographic densities of the extraction sites measured at T0 and T1, a significant enhancement was observed within the PRF group, but not within control group. The histological score decreased significantly from T0 to T1 within group PRF, but not within the control group; at T1, the PRF group showed a significantly lower histological score than the control group. These findings suggest that PRF could be able to stimulate the natural process of tissue healing and regeneration of post-extraction sites in dogs with spontaneous periodontal disease (PD).

## 1. Introduction

Periodontal disease (PD) refers to a group of plaque-induced inflammatory diseases that damage the tissues that hold the tooth, known as the periodontium (gingiva, periodontal ligament, cementum, and alveolar bone) [1].

Oral diseases are the most common pathology found during clinical examinations in dogs of all age ranges, and among these PD is the most frequently diagnosed. By just 2 years of age, 80% of dogs have some form of periodontal disease [2].

A 5-stage method based on a combination of oral examination and radiographic findings has been accepted by The American Veterinary Dental College (AVDC) and is most commonly used in clinical veterinary settings. In this model, Stage 0 represents healthy periodontal tissues and Stage I represents the reversible stage of gingivitis, where gums are inflamed but no bone loss has occurred [3]. While visible changes are associated with the progressive stages II–IV (mild, moderate, and severe PD), the most important defining element of these stages is bone loss and periodontal ligament involvement, which lead to gingival recession and periodontal pocket formation [4,5]. Periodontal bone loss is irreversible without regenerative therapy [2].

PD is diagnosed on the basis of clinical findings, periodontal probing, and dental radiography. As the degree of PD can vary by tooth within a mouth, treatment planning has moved in the direction of evaluating each tooth as a patient within the patient [6].

Platelet-rich fibrin (PRF) is an autologous hemo-component for topical use consisting of a fibrin mesh, in which platelets, leukocytes, and growth factors are concentrated, free of any added chemical substance [7,8,9,10]. The fibrin mesh has a complex tridimensional architecture that lets this biomaterial act as a scaffold for cells and new tissue deposition; it also sustains a significant slow release of growth factor for at least one week, stimulating prolonged tissue remodeling [11,12,13]. 

Since the first description by Choukroun in 2000 [14], PRF has become an important surgical adjuvant in oral surgical procedures. To date, this biomaterial has gained increasing attention in the field of dental research. Some of the possible recommendations for the use of this biomaterial in oral surgical procedures include third molar surgery, alveolar ridge preservation after tooth extractions, sinus lift procedure, repair of alveolar cleft, dental implants, surgical treatment of medication-related osteonecrosis of the jaw, and treatment of oro-antral communications [15,16,17,18,19,20,21]. 

Many studies evaluating the effect of PRF in alveolar post-extraction sockets have been carried out in humans. In most of these studies, the use of PRF was clearly beneficial [13,20,22,23,24,25,26,27,28,29,30], while in others the utility of PRF seemed to be more controversial, failing to show enhancement of the socket healing process [31,32,33]. In recent systematic reviews, definitive conclusions could not be drawn [34,35,36,37].

In dogs, PRF has been used in different fields, including skin wound healing, esophagotomy suture line reinforcement, bone healing, tendon healing, and articular cartilage repair [38,39,40,41,42,43,44]. 

In some studies, the PRF has been tested on the healing of canine extraction sockets and in peri-implant sites, however all of these studies were experimental; therefore, lesions and tooth extractions were specially created in healthy dogs, which were used animal models for preclinical purposes [45,46,47,48,49,50,51,52,53]. 

To the authors’ knowledge, this is the first randomized controlled trial evaluating the effects of autologous PRF in post-extractive alveolar sockets in dogs with spontaneous periodontal disease.

This study was planned to evaluate and compare wound healing and alveolar bone regeneration on dental extraction sockets with and without topical application of PRF in dogs. The hypothesis was that PRF would show positive effects on post-extraction sites in dogs compared with standard management. An assessment of the feasibility of PRF production and application procedures in canine extraction sites was also performed. 

## 2. Materials and Methods 

### 2.1. Inclusion and Exclusion Criteria

Dogs of different breeds, age, and sex showing grade III/IV periodontal disease (PD), who had an indication for bilateral extraction of maxillary or mandibular teeth simultaneously, were included in the split-mouth study. Informed owner’ consent was collected before enrollment. 

Dogs whose owners did not sign the informed consent, dogs indicated for traumatic extractions, dogs suffering from other local or systemic diseases (including malnutrition, cardiovascular, infectious, immunologic, and endocrine diseases), and dogs who had received topical or general therapies in the last 4 weeks (including chemotherapy, radiotherapy, hormonal therapies, corticosteroids) that could potentially influence the results of the study were excluded from the study, considering that the progression of periodontal disease is determined by the virulence of the bacteria combined with the host response [54]. 

### 2.2. Therapeutic Protocol

The protocol of the study was approved by the Committee in Charge of Animal Welfare of the University of Camerino (protocol number E81AC.10/A). 

#### 2.2.1. Preparation of Platelet-Rich Fibrin

For the preparation of PRF, the original Choukroun procedure was followed and specific materials were used (Process for PRF S.a.r.l, Nice, France) [7,14]. An autologous blood sampling was performed on each patient using a 21G butterfly needle connected to a 10 mL special glass vacuum A-PRF Plus (Advanced Platelet-Rich Fibrin Plus) tube, with no anticoagulant. A total 10–30 mL of blood (corresponding to 1–3 tubes) was collected from each dog on the basis of the number of planned extractions and types of dental sockets. The autologous blood was immediately centrifuged at 207× *g* for 8 min using the specific DUO centrifuge, following the manufacturer’s instructions. This procedure allows to obtain the advanced PRF plus. 

**Figure 1:**
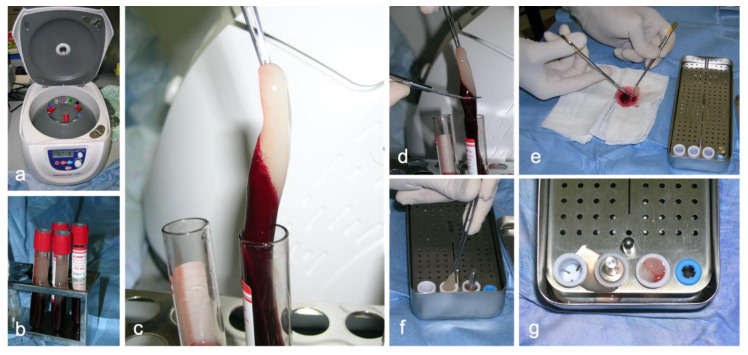
Platelet-rich fibrin preparation: (**a**) centrifugation of autologous whole blood using the specific DUO centrifuge; (**b**) Advanced PRF plus tubes immediately after centrifugation; (**c**) extraction of the PRF clot; (**d**) Coarse removal of the red blood cell layer; (**e**) careful isolation of the fibrin clot; (**f**) positioning of the fibrin clots in the specific wells of the PRF box; (**g**) squeezing of the fibrin clot using the metal piston to obtain a PRF plug from each well.

Five minutes after centrifugation, the fibrin clots were easily separated from the red blood cells using the sterilized instruments of the PRF set. A special PRF box was used to obtain PRF from the fibrin clots. To obtain PRF plugs, the fibrin clots were placed in the appropriate wells of the PRF box and squeezed with the weight of the metal piston to obtain a PRF plug from each well. The PRF plugs were then used for filling the extraction sites in the treatment group (Figure 1).

#### 2.2.2. General Anesthesia Procedure

The dogs were premedicated with dexmedetomidine (2 μg/kg) and methadone (0.2 mg/kg) mixed in the same syringe given intravenously through a catheter previously placed in the cephalic vein. The induction of general anesthesia was performed 10 min later with intravenous propofol (2 mg/kg). Following orotracheal intubation, anesthesia was maintained with isoflurane delivered in 100% oxygen. An appropriate dental nerve block technique was performed with 0.5% bupivacaine to interrupt the sensory stimuli locally during extraction.

#### 2.2.3. Surgery Procedure

All teeth with significant clinical mobility, greater than 50% attachment loss, or with furcation exposed were extracted.

Before oral procedures, the mouth was irrigated with chlorhexidine 0.12% solution and a pharyngeal gauze pack was used to prevent blood clots, fluid, or debris aspirations.

Unless teeth were severely mobile, surgical open technique was performed. In order to provide proper exposure of the alveolar bone supporting the teeth to be extracted, gingiva, mucosa, and periosteum were reflected. The gingiva was incised using a #15 blade inserted into the gingival sulcus. Then, a single vertical releasing incision was made and extended apical to the mucogingival junction in order to create a triangle flap. Once a mucoperiosteal flap was elevated and retracted, removal of the buccal alveolar bone was performed using a round diamond bur to visualize the furcation prior to sectioning multirooted teeth. A tapered bur on a high-speed handpiece was used to section the multirooted teeth, in which each root was separately extracted. A dental elevator placed in the periodontal space was used to loosen teeth prior to application of the extraction forceps. In some cases, a luxator was applied to cut Sharpey’s fibers within the periodontal ligament. The sharp bone edges present after extraction were removed by alveoloplasty. Following alveoloplasty, the empty alveolus was cleared of debris.

To address the research aim, the authors designed and implemented a split-mouth study, in which each patient also served as control: for each dog, the post-extraction alveolar sockets of one side of the mouth (left or right) were treated with the PRF (group PRF) and the sockets of the contralateral side underwent natural healing (group C). The side treated with PRF was randomly chosen through standard software (Microsoft Excel for Mac, version 16.36, Microsoft Office 365, Microsoft, Redmond, WA, USA). 

Post-extraction sockets allocated in the treatment side were filled with autologous PRF plugs, whereas the other sockets were left empty as control sites. 

**Figure 2:**
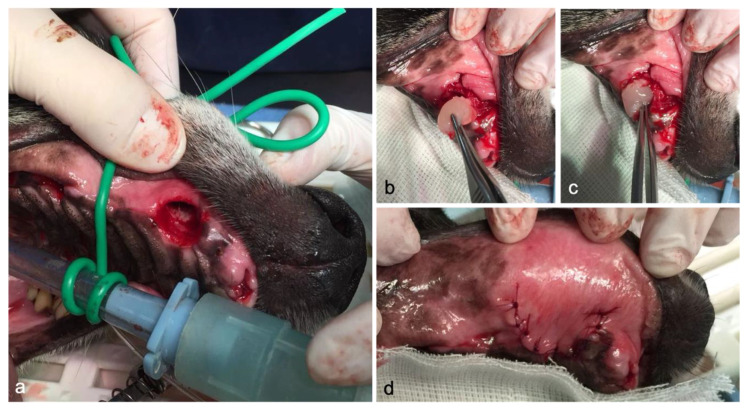
Surgery procedure and PRF application: (**a**) alveolar socket immediately after dental extraction; (**b**) application of PRF inside the post-extraction alveolar sockets; (**c**) post-extraction socket filled with autologous PRF plug; (**d**) apposition of mucogingival flap and suturing with monofilament material.

In both groups, the mucogingival flap was repositioned and then sutured with interrupted sutures using a 5–0 usp size monofilament material and a reverse-cutting, three-eights-circle needle (Figure 2).

#### 2.2.4. Post-Operatory Care

After surgery, each patient underwent oral administration of carprofen (2 mg/kg per day for 4–5 days) and amoxicillin and clavulanic acid (20 mg/kg every 12 h for 10 days), and was fed with soft canned food for at least 3 days. Postoperative pain was treated with intravenous buprenorphine (20 μg/kg).

### 2.3. Evaluation Protocol and Outcome Measures 

Clinical, radiographic, and histological assessments were performed at recruitment (T0) and after a three-week follow-up (T1). 

#### 2.3.1. Clinical Assessment

In each dog physical examination, hematological and hematochemical profiles and oral evaluation were performed. After the patients had been anesthetized, a complete oral examination, including tooth-by-tooth visual examination and gingival sulcus probing, was undertaken. 

During the oral examination, the gingivitis index, clinical attachment loss (gingival recession or probing pocket depth), bleeding on probing, plaque and calculus indices, furcation involvement, and mobility were highlighted to classify the PD stage (0–4). Abnormal findings from oral examinations were charted and recorded. 

#### 2.3.2. Radiographic Evaluation

All patients underwent a pre-operative radiographic study under general anesthesia to evaluate the alveolar bone condition, the continuity of the lamina dura, the width of the periodontal ligament space, and the configuration of the roots in order to plan the extraction treatment. 

Post-operative radiographs were taken immediately after surgery (T0) and after 3 weeks (T1) to evaluate the progress of the healing process, measuring the radiodensity of alveolar socket sites. All radiographic examinations were blindly performed. 

Each dog was placed in a lateral recumbency position on the X-ray table and slightly oblique lateral radiographic projections of the skull with extraoral technique (both right and left) were obtained in order to avoid the overlap of the two hemiarches. In each radiograph, the radiographic beam was centered on the mandibular or maxillary hemiarch under evaluation. 

To ensure the repeatability of the radiographic images, the exposure parameters used during shooting (intensity in mA, shutter speed in hundredths of a second, and electrical potential in kV), the degree of mouth opening, and the inclination of the head on the plane (head tilt) used at T0 were recorded and also used at T1. 

The degree of the head tilt determined the degree of obliquity of the radiographic projection. The appropriate inclination of the head on the radiographic plane was obtained using a radiolucent thick polystyrene wedge, a 60 cm long bubble level, and a base-supplied carpenter’s square. The radiolucent wedge was positioned between the dorsal–lateral portion of the head and the tabletop until reaching the desired head tilt that allowed an X-ray to be obtained, in which the alveolar sockets were clearly visible with no overlapping of the contralateral hemiarch. Once an appropriate X-ray was obtained, keeping the radiolucent wedge in position, the bubble level was placed onto the prominence of the zygomatic bone on the opposite side and kept parallel to the radiologic tabletop. The distance between the radiologic tabletop and the parallel straight-line tangent to the zygomatic process drawn by the axis of the bubble level was measured in millimeters using the square. This distance recorded at T0 was also used at T1, using the same radiographic procedure, with the aim of obtaining the same positioning and radiographic view.

To radiographically evaluate the degree of bone regeneration, Image J software (version 1.51) was used, which allows digital image processing to evaluate the bone density of the extraction sites, and consequently to indirectly trace the degree of bone regeneration. The X-ray images acquired in DICOM (Digital Imaging and Communications in Medicine) format were converted into TIFF (Tagged Image File format) format in 8-bit grayscale. The mineralization gradations of these images were then converted into a pseudo-color scheme of gradations at 16 equal intervals, obtaining an extension of the opacity levels (grey levels) in an optical density scale of pixels from 0 to 255. The average density of the extraction sites was measured by selecting the area of interest in the post-operative radiographic image (T0) and in the follow-up image (T1). 

#### 2.3.3. Histological Examination

Small bone and gingival biopsies were taken at T0 and T1 from both the extraction sites treated with PRF and from the control ones. Each biopsy sample was immersed in a 10% formalin-buffered solution and sent to the laboratory for histological examination. The investigator assigned a secret identification code to each sample so that a blinded histological assessment could be performed. The pathologist was blinded to the study group, treatment, and study time. Upon arrival in the laboratory, formalin-fixed tissue samples were dehydrated, paraffin-embedded, and serial 3-μm thick sections were prepared for each sample, which were then placed to adhere on electrostatically charged slides (Histoline, Milan, Italy) and placed to dry on a hot plate at 40 °C. Hematoxylin and eosin (H&E)-stained tissue sections from each dog were evaluated for histopathologic lesions using a LEICA DM 2005 microscope and a Retiga 2000R digital camera (QImaging, Surrey, BC, Canada). A special histological scoring system was used to semi-quantitatively assess the presence of inflammation (considering four items—the epithelium, dermis, periodontal ligament, and alveolar bone) and regeneration (considering three items—proliferation, neo-angiogenesis, and fibrin clots), as shown below. For each item listed above, the scores ranged from 0 (none) to 3 (high amount). The evaluation was performed on three fields at a magnification of 10×. The histological parameters concerning inflammation or periodontal involvement were evaluated as follows. For the epithelium, a score of 0 was assigned for normal conditions, where up to 10 leukocytes were observed; a score of 1 was assigned for the presence of some leukocytes (20–30) in transcytosis without epithelial suffering; a score of 2 was assigned for diffuse leukocytes infiltration (30–70) with signs of epithelial alteration or degeneration and microerosions; and a score of 3 was assigned for severe infiltration of leukocytes (>70) with epithelial erosion-lost or ulcerations. For the dermis of periodontal area, a score of 0 was assigned for normal conditions, where up to 20 leukocytes were observed; a score of 1 was assigned for the presence of some leukocytes (30–50) in transcytosis or in the perivascular area; a score of 2 was assigned for diffuse leukocyte infiltration (50–90) with signs of perivasculitis and collagen shrinkage; and a score of 3 was assigned for severe infiltration of leukocytes (>90) with areas of necrosis. For periodontal ligament (perialveolar fibrous connective tissue), a score 0 was assigned for normal findings, without infiltration or edema; a score of 1 was assigned for the presence of some leukocytes (10–15) in the perivascular area; a score of 2 was assigned for diffuse infiltration of leukocytes (20–40) with signs of shrinkage or fragmentation of connective tissue; and a score of 3 was assigned for severe leukocyte infiltration (>40) with ligament destruction. For alveolar bone, a score of 0 was assigned where bone fragment was normal; a score of 1 was assigned for activation of some osteoblasts or osteoclasts; a score of 2 was assigned for diffuse bone resorption; and a score of 3 was assigned for severe bone resorption with polynucleated giant osteoclasts. 

In an attempt to characterize the inflammatory infiltrate, an adjunctive parameter was considered for each inflammatory score: polymorph nuclear cells (PMNs) and mononuclear cells (lymphocytes, plasm cells, and macrophages MNs) were counted, scored, and expressed as a value derived from the PMN/MN ratio. The values of this ratio ranging between 0 and 1 were considered to express chronic inflammation (with a heavy predominance of MNs); a value 1 was considered an expression of a chronic-active (or pyogranulomatous) inflammation (with a similar number of PMNs and MNs); and the values >1 were considered typical expressions of an acute inflammation (with a predominance of PMNs).

The histological parameters concerning regenerative aspects were evaluated using a ×40 objective, a ×10 eyepiece, and a square eyepiece graticule (10 × 10 squares, having a total area of 62,500 μm^2^). Ten appropriate fields were chosen for each compartment and arithmetic means were calculated for each bioptic sample. Results were expressed as mesenchymal cells (mitotic fibroblasts or osteoblasts)/micro vessels per 62,500 μm^2^, which were evaluated as follows. For fibroblast proliferation, a score of 0 was assigned for normal connective tissue (fibrocytes without mitosis); 1 for some sporadic mitotic figures in fibroblasts (1–2 mitosis/high power fields, HPFs); 2 for moderate activation with diffuse presence of mitotic fibroblasts (3–5 mitosis/HPFs); and 3 for diffuse fibroblastic activation (>5 mitosis/HPFs). For neoangiogenesis, a score of 0 was assigned in the case of the absence of neo-vessel formation; 1 for few and interspersed neo-vessels (1–2); 2 for moderate neo-vessel formation (3–5); and 3 for diffuse presence of neo-vessels (>6 well-formed capillaries). 

For the presence of the fibrin clot, a score of 0 was assigned in the case of absence; 1 for fragments of amorphous material; 2 for well-preserved clot fragments; and 3 for a well-preserved clot.

The final score (ranging from 1 to 22) was obtained by subtracting the total score of the regeneration parameters (regeneration score, R.S.) from that of inflammation (inflammation score, I.S.), adding 10 points to this difference as specified by the following formula:

*Final Score* = (I.S. − R.S.) + 10 (1)

A low final score indicates good regeneration, while a high final score indicates poor regeneration or high inflammation. 

### 2.4. Statistical Analysis

Radiographic and histological data were presented as the mean, median, and standard error of the mean.

Cardinal data were assessed for normality using the D’Agostino–Pearson omnibus K2 test.

Radiographic density was compared between the two groups and within each group from T0 to T1 using a paired *t*-test. Histological scores were compared between the two groups and within each group from T0 to T1 using the Wilcoxon matched-pairs signed rank test. 

To account for potential clustering effects deriving from multiplicity of outcome measurements from teeth (observational units) within each split-mouth side (experimental unit), the analysis was performed at the cluster level after a summary outcome measurement calculation per cluster was performed. 

Difference with *p*-values <0.05 were considered statistically significant. All data were analyzed with the software GraphPad Prism 8 for MacOS, version 8.2.1 (GraphPad software Inc., San Diego, CA, USA). 

## 3. Results

This study was carried out on 12 dogs of both sexes, aged between 10 and 15 years. A total of 62 post-extraction alveolar sockets were considered as observational units in the study, representing 31 pairs of sockets symmetrically occurring in each of the sides of the mouths. In each dog, each side represented the clustered experimental unit randomly allocated to the treatment group (group PRF) or control group (group C), with a mean of 2.58 sockets per cluster (5.6 sockets per dog). 

### 3.1. Clinical Findings

**Figure 3:**
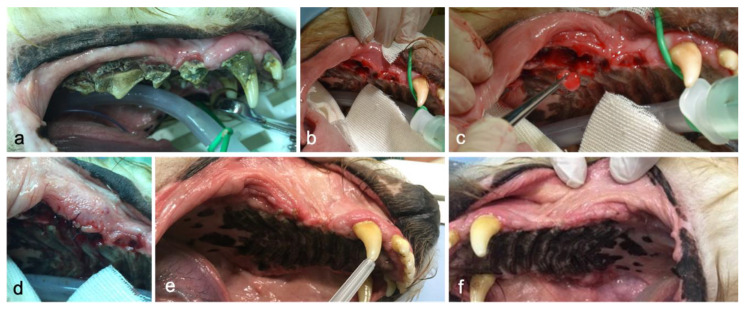
Clinical appearance in various stages of treatment and evaluation protocols: (**a**) pre-operative condition at recruitment; (**b**) intraoperative condition of post-extraction sockets; (**c**) intraoperative condition, application of PRF in the treated side (group PRF); (**d**) post-operative condition immediately after suturing of the mucogingival flap; (**e**) split-mouth treated side (group PRF) after a three-week follow-up (T1); (**f**) split-mouth control side (group C) after a three-week follow-up (T1).

At recruitment (T0), all dogs showed severe PD classified as grade 3 or 4; grade 2 or 3 gingivitis; gingival retractions with evident exposure of root cement and furcation in multiroot teeth; and strong halitosis due to high accumulation of plaque and tartar. In some cases, the periodontitis was complicated by severe gingival hyperplasia. The gingival probing revealed the presence of evident periodontal pockets and a grade 2 or 3 of furcation exposure in multiroot teeth. Surgeries were performed with no major complications. Three weeks after dental extraction (T1), all patients underwent again clinical examination of the oral cavity, during which no complications were observed, nor were clinically evident differences observed between the treated sites and the control sites. During biopsy procedures at T1, higher dissection difficulties were found in the treated sites due to adhesion of the gingiva to the underlying alveolar bone (Figure 3).

### 3.2. Radiographic Findings

**Figure 4:**
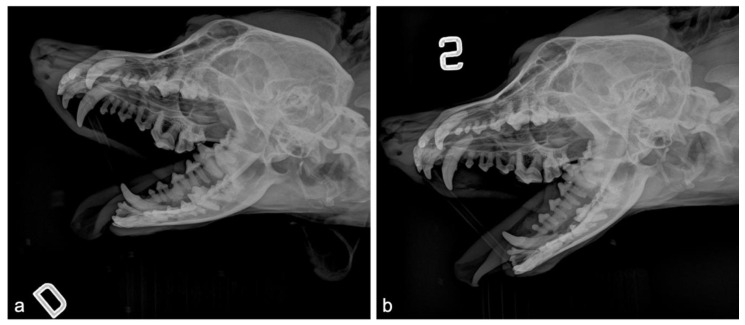
Pre-operative radiographic study: (**a**) oblique projections in the right lateral recumbency (D indicates the right lateral recumbency), left maxillary hemi-arch, and right mandibular hemi-arch are visible inside; (**b**) oblique projections in the left lateral recumbency (S indicates the left lateral recumbency), right maxillary hemi-arch, and left mandibular hemi-arch are visible inside.

A pre-operative radiographic study of the dental elements intended for extraction showed periapical bone resorption greater than 50% and evident root resorption (Figure 4).

**Figure 5:**
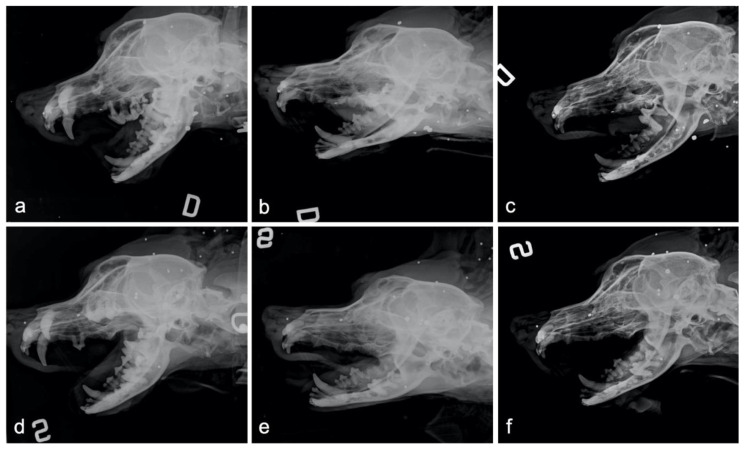
Time point radiographic sequence of both sides during the split mouth study. The first column (**a**,**d**) shows the preoperative radiographic aspect at recruitment. The second column (**b**,**e**) shows the radiographic aspect of the immediate postoperative time (T0). The third column (**c**,**f**) shows the radiographic aspect after three-week follow-up (T1). The first line (**a**–**c**) shows the oblique radiographs obtained for the dog in the right lateral recumbency (D indicates the right lateral recumbency), left maxillary hemi-arch, and the right mandibular hemi-arch are visible inside. The second line (**d**–**f**) shows the oblique radiographs obtained for the dog in the left lateral recumbency (S indicates the left lateral recumbency), right maxillary hemi-arch, and left mandibular hemi-arch are visible inside.

This systematic positioning procedure gave excellent repeatability for the radiographic projections, with perfectly superimposable images obtained for the different study times: the images at T0 and T1 were suitable for a reliable clinical radiographic interpretation of findings and measurements, reducing the possibility of positioning bias (Figure 5).

The mean percentage of variation of the radiographic density in extraction sites from T0 to T1 was +23.86% in group PRF and +12.12% in group C. However, the difference between the two groups was not statistically significant at T0 (t = 0.05901, df = 11, *p* > 0.05) or at T1 (t = 1.106, df = 11, *p* > 0.05). 

Within group PRF, comparing the radiographic densities of the extraction sites measured at T0 (70.694 ± 8.212) and T1 (87.562 ± 10.101), a significant difference was observed (t = 2.622, df = 11, *p* = 0.0237).

**Figure 6:**
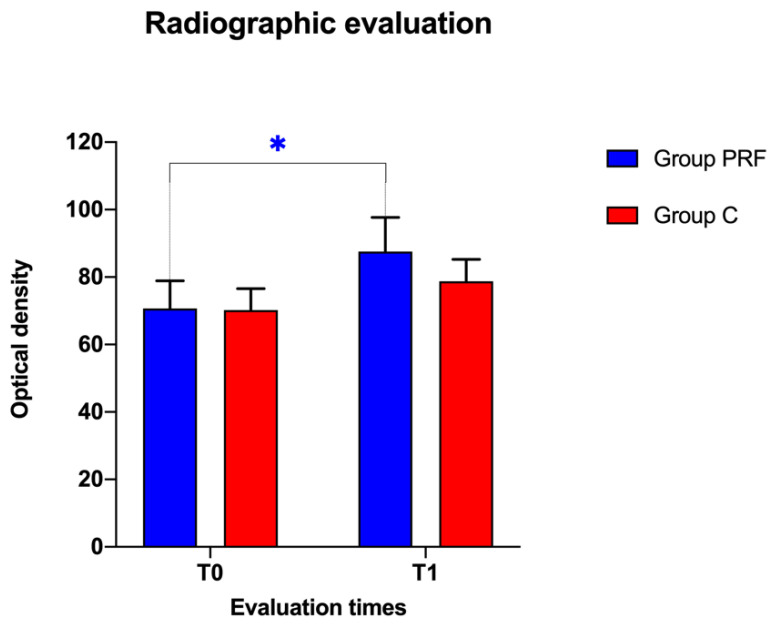
Bar plot showing the means ± standard errors of radiographic optical densities of post-extraction sockets in the treated group (Group PRF) and control group (Group C) at recruitment (T0) and after a three-week follow-up (T1). The blue asterisk indicates statistical significance within group PRF from T0 to T1. * indicates statistical significance within group PRF from T0 to T1.

Within group C, there were no significant differences in radiographic densities (t = 2.156, df = 11, *p* > 0.05) between T0 (70.222 ± 6.358) and T1 (78.730 ± 6.524) (Figure 6).

### 3.3. Histological Findings

**Figure 7:**
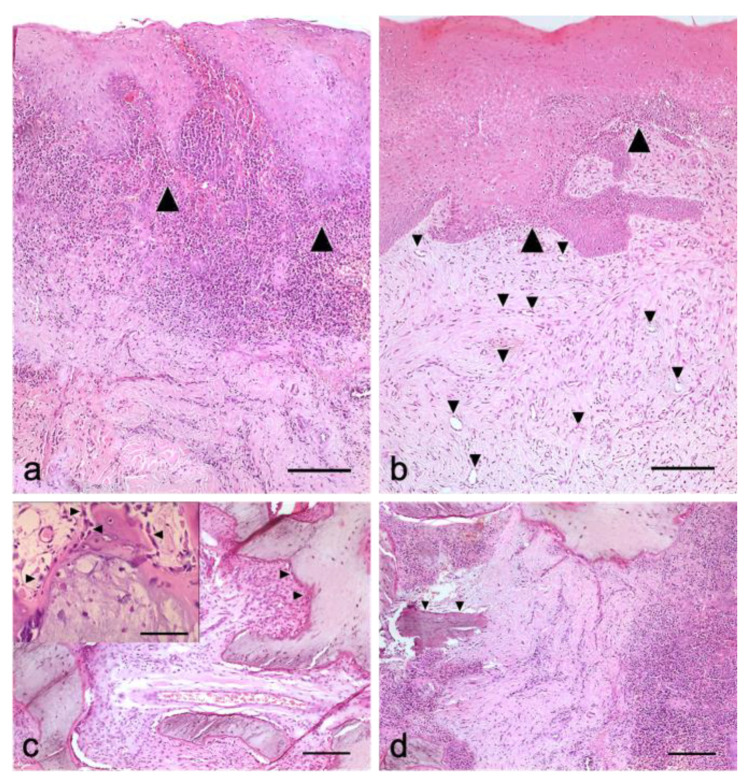
Histological photomicrograph of hematoxylin and eosin (H&E)-stained sections of bioptic samples from a post-extractive surgical wound, three weeks after the surgery: (**a**) untreated sample from control group shows an abundant infiltration of inflammatory cells in the epithelial layer (arrow heads), with an irregular epithelial layer, micro-hemorrhages, and extensive edema, indicating poor repair parameters; (**b**) surgical site treated with PRF reveals scant inflammatory cells with epithelial hyperplasia (big arrow heads) and new collagen synthesis with irregular orientation, which is associated with remarkable neo-angiogenesis (arrow heads); (**c**) biopsy sampled at the level of the periodontal bone from a surgical site treated with PRF evidences ontogenetic processes (arrow head), with some new osteoid-synthetizing basophilic osteoblasts with large activated nuclei (insert, arrow heads); (**d**) bone sampled from a surgical site in which no treatment was applied—a moderate persisting inflammatory response with some fragments of bone being resorbed by osteoclasts is indicated by the arrow head. H&E, scale bar = 400 µm (In insert, bar = 250 µm).

Histological analysis of bioptic samples belonging to untreated post-extractive alveolar sockets from the control group revealed abundant infiltration of inflammatory cells in the connective tissue (inflammatory scores between 2 and 3, with PMN/MN ratios of between 1 and 3), and particularly in gingival epithelium as exocytose, limited amounts of vessels buds, extensive edema, and poor repair parameters three weeks after surgery (Figure 7A). However, for post-extractive wounds treated with PRF, at the same time point a relatively moderate inflammatory response with diffused chronic inflammatory cells (I.S. between 1 and 2, with PMN/MN ratios ≤ 1) among the gingival epithelial rete ridges and in connective tissue were observed (Figure 7B). In this group, we observed poor signs of inflammatory response in the connective tissue, with sparsely scattered chronic inflammatory cells (i.e., lymphocytes, scattered plasm cells, and activate macrophages; I.S. 0 to 1, PMN/MN ratio between 0 and 1), dense accumulation of relatively well-aligned new collagen fibers, and relatively complete integrity between the epithelial cells with moderate thickness. In biopsies belonging to PRF-treated post-extractive wounds, a marked proliferation of fibroblasts synthetizing new collagen with irregular orientation was also observed (Figure 7B). Our study demonstrated that neovascularization was more prominent in regions treated with PRF after dental extraction in comparison with the control group (Figure 7B). On the other hand, the amount of inflammatory parameters, particularly edema, was marginally higher in untreated control sites compared with PRF-treated post-extractive wounds. Abundant new vessels, buds, and collagen synthesis directly below the gingival epithelium were apparent in the region where PRF was applied. However, post-extractive control wounds showed mildly proliferated epithelial cells on the wound surface. In the histological survey, after three weeks we observed moderate to severe chronic inflammatory cell accumulation (I.S. between 2 and 3, with PMN/MN ratios between 0 and 1) in the control group, particularly in the *lamina propria* directly below the gingival epithelium and deep in the connective tissue, with moderate tissue disruption, edema, and weak scaffolding of collagen fibers, which generally indicated relatively poor healing that seemed to be strongly reduced compared with PRF-treated post-extractive wounds. 

In addition, biopsies sampled at the level of the periodontal bone showed some new osteoid synthetizing basophilic osteoblasts with large activated nuclei (Figure 7C, insert) in post-extractive wounds treated with PRF (Figure 7C) in comparison with those surgical sites where no periodontal treatment was applied, and in which a moderate persisting inflammatory response with very few repair parameters was observed (Figure 7D). 

The mean histological scores decreased in both groups during the study. The percentages of variation of the histological scores from T0 to T1 were −60.23% in group PRF and −9.29% in group C. The difference between groups was not statistically significant at T0 (W = −40.00, *p* > 0.05), but it was significant at T1 (W = 78.00, *p* = 0.0005) 

Within group PRF, comparing the histological scores measured at T0 (14.667 ± 1.144) and T1 (5.833 ± 0.601), a significant difference was observed (W = −78.00, *p* = 0.0005). 

**Figure 8:**
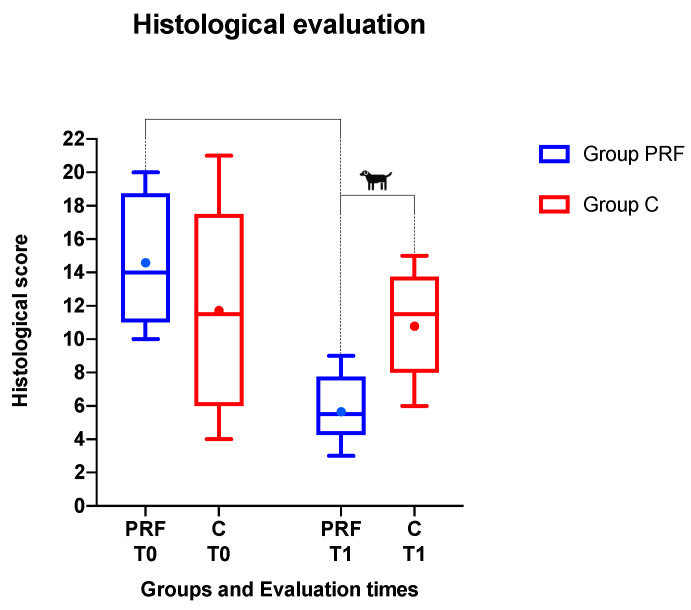
Boxplot showing the histological scoring system in the treated group (group PRF) and in the control group (group C) at recruitment (T0) and after a three-week follow-up (T1). The ends of the whiskers show minimum and maximum score values; boxes show the median, the first quartile, and the third quartile; red and blue dots show the mean. The blue asterisk indicates statistical significance within group PRF from T0 to T1. The black dog indicates statistical significance between groups at T1.

Within group C there was no significant difference in histological scores (W = −30.00, *p* = 0.2505) between T0 (11.667 ± 1.772) and T1 (10.583 ± 0.933) (Figure 8).

## 4. Discussion

Beneficial effects of autologous PRF in post-extractive alveolar sockets in dogs with spontaneous periodontal disease were appreciated for the first time in a randomized controlled trial. The decision to perform this clinical trial in spontaneous and not in experimentally-induced injuries was primarily for ethical reasons, but at the same time it allowed reliable assessment of the clinical efficacy of PRF in the conditions in which it would be most appropriate—the spontaneous periodontal disease, an impaired condition that is extremely difficult to reproduce experimentally. On the other hand, the experimentally induced injury in live animals is considered an acute injury performed on a healthy tissue, which is fully able to react to injuries. In this context, the evaluation of spontaneous pathology in dogs, in addition to having a clinical value for the canine species itself, could have a high translational value for human medicine. 

A split-mouth design was applied in this study. This design has the advantage that participants act as their own controls, as both interventions are applied within the same patient, meaning much of the inter-subject variability is removed, resulting in increased study power for the same number of participants compared with a parallel-design in which participants receive only one intervention [55]. The application of strict inclusion criteria allowed the adequate uniformity in the sites of each dog required for this type of study design, decreasing the risk of selection bias. In this context, this design provided the best possible control group because both treatments were carried out in the same dog, by the same surgeon, and applying the same surgical procedure, with identical microbiologic and environmental conditions, as reported in other studies [56]. Since multiple observation units were obtained in the same subject, the authors identified the experimental unit for randomization and analysis at the cluster level, constructing a summary statistic for each cluster to account for clustering effects, as recommended by the literature [55,57]. The occurrence of a carry-across effect could be considered a limitation in the application of the split-mouth design [55]. Potential carry-across effects were not expected in this study, since PRF therapy is topically applied, taking place inside the post-extraction socket, meaning it cannot in any way influence the healing of the sockets of the contralateral cluster group. 

Numerous studies have shown that PRF is able to stimulate and accelerate healing and tissue regeneration; some advantages over other platelet concentrates have also been reported [9,25,30]. According to a recent systematic review including in vitro, in vivo, and clinical studies, the currently available literature supported the use of PRF for soft tissue regeneration, wound healing, and angiogenesis, although a certain heterogeneity of results and lack of appropriate controls were observed in the majority of the studies. Despite this, the most frequent emerging result is the ability of PRF to stimulate the regeneration of a wide range of tissues by encouraging the recruitment and proliferation of numerous cell types, including endothelial cells, keratinocytes, and dermal and gingival fibroblasts. All this happens thanks to the presence of cytokines and growth factors trapped in the fibrin network from autologous sources [58].

The second intention healing of the post-extraction site is an important model for evaluation of the regenerative capacities of PRF with respect to both bone and soft tissue. The clinical application of PRF in bone defects and extraction sites is also related to its ability to function as a “competitive barrier”; as the soft tissue regenerates faster than the hard tissue, the use of this hemo-component allows the periosteum to be strengthened by acting as an interface between these two types of tissues, avoiding the migration of the soft tissue at depth inside the bone defect [59]. In particular, in a study carried out in dogs, the ability of the PRF to prevent the migration of soft tissue within the extraction site when it was positioned around the implant was highlighted, demonstrating the potential of PRF to act as a physical barrier to avoid soft tissue downgrowth in the socket, resulting in increased bone formation [49]. 

In this study, advanced PRF plus (A-PRF plus) used, which is classifiable among leucocyte- and platelet-rich fibrin (L-PRF) or Choukroun’s PRF [8]. To obtain A-PRF plus, autologous venous blood is placed in dry glass tubes subjected to a single-step centrifugation at a lower speed and for a shorter time than the original standard method (S-PRF) [60]. This procedure seems to allow better distribution and content of autologous cells, including platelets, neutrophils, and macrophages, by better exploiting their potential regenerative properties [60].

Some laboratory studies were carried out on platelets and leukocyte contents, and on the amount of GFs in both standard and advanced PRF in humans [11,12,60,61,62]. Several studies have reported the amount of GFs in canine PRF; however, to the authors’ knowledge, a complete characterization of the canine PRF has not yet been performed [46,52,63]. 

The procedure for PRF preparation did not entail any difficulties or problems; it was very rapid and feasible in a clinical veterinary setting. It did not require the collection of large quantities of autologous blood, therefore it could also be used in small dogs. The only critical point observed during the study, as indicated by literature [7], was that whole blood needs to be immediately processed after collection, since the procedure does not involve the use of any anticoagulants or gelling agents. In the absence of anticoagulants, platelet activation and fibrin polymerization are triggered quickly [8]. For this reason, the potential of the PRF could be closely related to the time elapsed between blood sampling and centrifugation; it may also be important not to extend the time from preparation to application [9]. No other disadvantages or side effects were shown during the study or described in the literature. 

Clinical follow-up showed no significant differences between the treated sites and the control sites. In both groups, a reduction in the degree of periodontitis or complete healing was observed, thanks to the extractive treatment in synergy with the natural regenerative capacities.

However, an important though not objectifiable finding was detected intraoperatively during the small dissection of the mucogingival flap, which is necessary to perform the biopsy sampling at T1—greater difficulty in separating the mucogingival tissue from the underlying alveolar bone was observed in the PRF-treated sites compared to the control sites. This finding could be interpreted as greater adhesion of the mucogingival tissue to the alveolar bone, potentially due to PRF action.

Comparing the radiopacity of the extractive sites between the two groups during the study, the PRF group showed a higher increase than the control group, even if this difference was not statistically significant.

Within both groups, an increase in the bone density of the extractive sites was observed during the study; however, in the PRF group this increase was statistically significant, while no statistical difference was detected in the control group. 

Although computed tomography could have been more accurate in detecting subtle differences in bone radiopacity, this study showed that the presence of the PRF induced an increase of the radiographically detectable density of the extraction sites; therefore, it could be assumed that when used in dental extraction surgery, PRF may positively influence the bone regeneration of the treated sites, as reported by other studies [15,19,20,44]. 

The histological evaluation revealed a clear difference between the extraction sites treated with PRF and the control sites; in particular, in the PRF group there was a reduction in inflammation and a marked tissue regeneration, while in the control group the final histological score showed a slight tendency to deteriorate. In the PRF group, we observed a remarkable decrease in the amount of the inflammatory parameters within the three weeks after surgery compared to those of the control group, suggesting that silver PRF altered or compressed the inflammatory events in the wounds and facilitated the early phases of wound healing. In addition, the ability of PRF to modulate cytokine production, and thereby to diminish the inflammatory response following burn injury (confirmed due to decreased inflammatory infiltration within the wound and a reduced PMN/MN ratio), was thought to contribute to its ability to promote and accelerate post-extractive wound healing and reduce secondary infections. On the other hand, the amounts of matrix and osseous regenerative parameters, particularly new vessels, collagen, and osteoid formation, were relatively higher in sites treated with PRF in comparison with biopsies belonging to control sites. Our result suggested that post-extractive wounds treated with PRF in general terms had all the hallmarks of relatively complete healing three weeks after the operation.

## 5. Conclusions

Both radiographic and histological findings agree in attributing to the PRF a potential ability to stimulate the natural process of tissue healing and regeneration within the extraction sites. Our findings suggest that PRF could be considered as an effective, simple, and cost-effective therapeutic support for the management of extraction sockets in dogs. Further studies will be necessary to confirm these results in wider trials and to evaluate the advantages of PRF compared to other treatments.
